# Effect of Resistance Training Methods and Intensity on the Adolescent Swimmer's Performance: A Systematic Review

**DOI:** 10.3389/fpubh.2022.840490

**Published:** 2022-04-04

**Authors:** Wei Guo, Kim Geok Soh, Noor Syamilah Zakaria, Mohamad Taufik Hidayat Baharuldin, Yongqi Gao

**Affiliations:** ^1^Department of Sports Studies, Faculty of Educational Studies, Universiti Putra Malaysia, Seri Kembanga, Malaysia; ^2^Department of Sports Studies, Faculty of Physical Educations, Ningxia Normal University, Guyuan, China; ^3^Department of Counselor Education and Counseling Psychology, Faculty of Educational Studies, Universiti Putra Malaysia, Seri Kembanga, Malaysia; ^4^Department of Preclinical, Faculty of Medicine and Defence Health, Defence University of Malaysia, Kuala Lumpur, Malaysia

**Keywords:** physical activity, speed, combination, distance, strength

## Abstract

**Background:**

Resistance training has been widely used in various sports and improves competition performance, especially in swimming. Swimming performance is highly dependent on muscle strength, especially short distances. For adolescent athletes, the existing literature has bound to prove that resistance training is undoubtedly bound to improve swimmers' performance.

**Objectives:**

This study adopts a systematic literature review to (1) examine the effects of resistance training on the performance of adolescent swimmers, and (2) summarize their training methods and intensity.

**Methods:**

The literature search was undertaken in five international databases: the SCOUPS, PubMed, EBSCOhost (SPORTDiscus), CNKL, Web of Science. The searches covered documents in English and Chinese published until 30th December 2020. Electronic databases using various keywords related to “strength training” and “adolescent swimmers” were searched. Sixteen studies met the inclusion and exclusion criteria where the data was then systematically reviewed using the PRISMA guideline. Furthermore, the physical therapy evidence database (PEDro) scale was used to measure each study's scientific rigor.

**Results:**

This review found that to improve the swimming performance of adolescents, two types of resistance training were used, specifically in water and on land, where both types of training can improve swimming performance. In addition, training with two types of resistance machines were better in the water than with one equipment. Resistance training can improve the swimming performance of adolescent swimmers at 50 m, 100 m, 200 m and 400 m distances. However, most studies only focused on the swimming performance at 50 m and 100 m lengths. A low-intensity, high-speed resistance training programme is recommended for adolescent swimmers to obtain the best training results.

**Conclusion:**

Water or land resistance training can improve the swimming performance. Given that both types of exercises have their strengths and weaknesses, combining these methods may enhance the swimmers' performance. In addition, despite the starting and turning phases consuming up to one-third of the total swimming time for short distances, literature in this area is limited.

**Systematic Review Registration:**

https://www.crd.york.ac.uk/prospero, identifier: CRD42021231510.

## Introduction

Swimming differs from other sports as it requires a particular environment involving water. Swimming is a systemic exercise that necessitates the coordination of the upper and lower limbs to ensure that the body performs its best in water (1). Building their support platform in water is, hence, essential among swimmers. The core to a solid and stable swimming movement is connecting the upper and lower limb movements in water while producing a stable support foundation (2, 3). Studies have found that swimming speed is the product of stroke rate and stroke length, where increasing either one of these would improve a swimmer's performance (4–6). It has also been suggested that improving swimming speed requires high frequency, duration and intensity, where a high total training volume may be expected (7).

Resistance training is defined as the ability of a given muscle, or group of muscles, to generate muscle strength under specific conditions (8, 9). Furthermore, resistance training has been found to increase the maximum muscle strength, thereby increasing the speed of strength development (10). It is generally believed that swimming performance is highly dependent on the muscle strength (11–15). The purpose of resistance training is to overload the muscles used in swimming and increase the maximum strength output. Resistance training has many physiological benefits including increased phosphate stores, contractile proteins, anaerobic power output, muscle structure, fiber bundles, protein synthesis, tissue remodeling and fast-twitch muscle fiber hypertrophy (16–19).

Furthermore, swimming performance is highly dependent on muscle strength (11, 20–22). The ability to exert force in water is the decisive factor, especially for short distances (23, 24) as many studies have described the importance of muscle strength of arms and legs and its generated force in swimming performance (25, 26). As compared to traditional resistance training (e.g. barbells, dumbbells, pull-ups, leapfrogs), elastic bands, Swiss balls, drag parachutes and resistance gloves are currently more commonly used to build strength. Research has shown that resistance training in either water or land is superior to traditional strength training (27–31). Where muscle strength is considered the primary determinant of competitive swimming success (32). In order to move freely in water, swimmers are required to overcome these resistance by continuously improving their strength (33).

Youth sports coaches tend to implement structured training methods based on college or professional models for adult athletes (34). This is due to the assumption that these methods would be similarly effective and applicable among adolescent athletes. However, the intensive training model among adults usually include year-round arrangements, professional training and daily physical exertion. Given that physical recovery during intense training is limited regardless of age and development (35, 36), most studies have shown that the response of prepubertal children to training is similar to that of mature athletes, but with a different degree (37–40). Hence, while resistance training can be used with adolescents, it should be conducted at a smaller intensity.

The present study is to specify a set of descriptions of swimming training methods, intensity, number of groups, repetitions or practice times for teenagers suitable for different research objects. Some studies have prescribed a set of suitable training methods for their subjects. The purpose of this paper is to find the law of resistance training for teenagers and sum up a set of principles and methods suitable for all teenagers swimming training. Therefore, the purpose of this systematic literature review is to (1) examine the effect of resistance training on the performance of adolescent swimmers and (2) summarize the training methods and intensity of adolescent swimmers.

## Methods

### Literature Search Strategy

The literature search was undertaken in five international databases: the SCOUPS, PubMed, EBSCOhost (SPORTDiscus), CNKL, Web of Science. The searches covered documents in English and Chinese published until 30th December 2020. Only articles in journals were accepted. The following electronic sources were searched: Science and China National Knowledge Infrastructure (CNKI). The key terms used were: (“weight exercise” OR “strength training” OR “strength exercise” OR “weight training”) AND (“adolescent swimmer” OR “juvenile swimmer” OR “teenager swimmer” OR “youth swimmer” OR “young swimmer” OR “junior swimmer” OR “children swimmer”). In addition, age (children and adolescents of 6–20 years in age) was used as the limiter.

### Inclusion and Exclusion Criteria

Used the PICOS (population, intervention, comparison, outcome, study designs) criteria as the inclusion criteria, is presented in Table 1. Only records presenting resistance training on aspect of performance of adolescent swimmer were included. Thus, studies were included if they met the following criteria: (1) A full text, peer-reviewed study published in English, describing the use of athletes (male and female) to explore the effects of resistance training interventions on swimming performance., randomized controlled trial (RCT), non-randomized controlled trial (Non-RCT) with two or more groups, and single-group trials with pretest and post-test design; (2) In this study, only included studies on planned and organized resistance training intervention to improve swimming performance; (3) Investigate the effects of resistance training on swimming performance athletes and assess at least one swimming performance, component outcome; (4) There were no restrictions on the sample size, study location, and intervention time for the included studies.

**Table 1:** Inclusion criteria according to the PICOS conditions.

**Items**	**Detailed inclusion criteria**
Population	Athletes
Intervention	Resistance training
Comparison	Two or more groups
Outcome	Swimming performance
Study designs	RCT or Non-RCT

The inclusion criteria were (1) swimming performance as an outcome measure, (2) swimmers had undergone structured swimming training programmes, and (3) participants were 20 years old and below. On the other hand, the exclusion criteria were (1) untrained, novice, masters, and paraplegic swimmers, (2) triathlon and water polo athletes, and (3) injured swimmers.

### Study Selection

**Figure 1:**
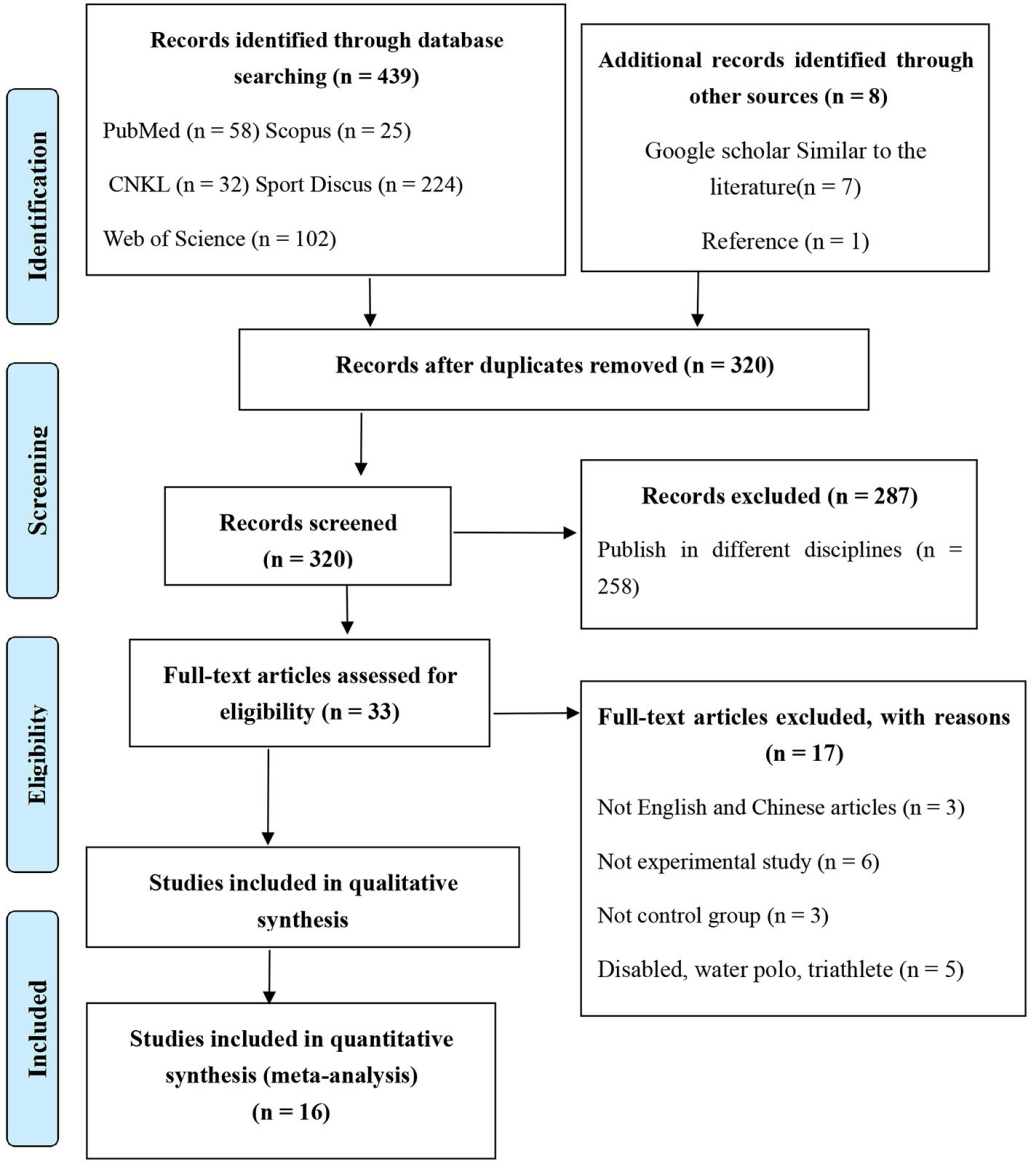
PRISMA flow chart of the study selection process.

This study used projects for systematic reviews and meta-analysis (PRISMA) guidelines to conduct this systematic review (41). The retrieval process, as shown in Figure 1, included an evaluation hierarchy that evaluated studies first by journal title, second by abstract, and third by full-text review where journal articles were selected according to the inclusion and exclusion criteria. An initial search yielded 439 published papers and after deletion of duplicates, 320 papers remained. In total 264 papers were further excluded as they were published in different disciplines while another 20 papers were deleted as the full text was unavailable. The remaining 33 papers were on the influence of resistance training on adolescent swimming performance. In total 17 papers were further excluded as they were neither in English nor Chinese, non-experimental studies, did not have a control group and included water polo, disability and triathlon athletes. As a result, 16 papers were included in this systematic review.

### Data Extraction and Quality Assessment

After the data search was complete, data were obtained from eligible studies in a predetermined extraction form [Including, (1) Author, title, publication year; (2) Research design; (3) Sample size, control group; (4) Participant characteristics (age, gender,etc.); (5) Intervention features (type, length, and frequency); (6) Measures index; and (7) Research outcomes]. One author abstracted information into the standard form and the other author checked it.

The Physiotherapy Evidence Database (PEDro) scale was subsequently used to assess the quality of the final 16 records (15, 33, 42–55). The PEDro scale was used to score the literature quality based on 15 items related to scientific rigor, which includes eligibility criteria, random allocation strategy, type of intervention, main exercises, training arrangements, test items, inter-group analysis, and results (56). If the study meets the criteria, the scale is scored with 1 point, but zero points were awarded if a criterion is not met. Research with a score of either 9 or 10 on the PEDro scale is considered methodologically excellent while a score of between 6 and 8 is good. Research with a 4 or 5 score is considered average, with scores lower than 4 suggesting that the research was methodologically poor. The authors rated all studies based on this scale as reported in Table 2.

**Table 2:** Population, study design and PEDro scale.

**Study**	**Population**	**Study design**							**Score**
		**Duration** **(weeks)**	**Frequency**	**Randomized**	**Intervention type**	**Main exercises**	**The training arrangement**	**Test project**	
Huang (42)	Young swimmers (13)	4	6 per week	YES	Water resistance exercises	Five meters of rubber tension band	8*30 s	50 m freestyle 400 m freestyle	6
Zhao (33)	Young male swimmers (11–15)	8	3 per week	YES	Land resistance training	Prone Swiss ball, pull-up, Isodynamic tension. resistance sprints.	3*30 times 5*60 times 3*30 m	100 m freestyle 100 m breaststroke, 100 m butterfly, 100 m backstroke	6
Batalha et al. (43)	Young swimmers (12-15)	10	8h of per week	YES	Water and land	Elastic resistance bands.	3*30 s 4*30 s 5*30 s	Shoulder Rotator Cuff Strength and Balance	6
Dalamitros et al. (44)	Young swimmers (14.82 ± 0.45)	24	6 per week	NO	Land resistance training	Swim resistance machines	5 to 7 km	Concentric knee extension and flexion peak torque	6
Amaro et al. (45)	Young Swimmer (12.7 ± 0.7)	10	2 per week	YES	Land resistance training	Dumbbell 1.5 kg Russian twist 3 kg Push-up	2*30 times 2*40 times 2*90 times	Vertical jump, ball throwing 50 m freestyle	6
Naczk et al. (46)	Young swimmers (15.8 ± 0.4)	4	3 per week	YES	Land inertial training	ITMS inertial training measurement system	2*60 times	100 m butterfly 50 m freestyle	6
Marques et al. (47)	Young swimmers (16.6 ± 0.7)	20	2 per week	NO	Land strength training	Full squat bench press jump height	30–40% 1RM	50 m freestyle	6
Girold et al. (15)	Young swimmers (16.5)	12	2 per week	YES	Land strength training	Assisted-sprint exercises	80–90% 1RM	50 m freestyle	6
**Study**	**Population**	**Study Design**							**Score**
		**Duration** **(weeks)**	**Frequency**	**Randomized**	**Intervention type**	**Main exercises**	**Frequency**	**Test project**	
Toussaint et al. (48)	Young swimmers (18.50 ± 3.30)	10	2 per week	YES	Water resisted training	System to measure active drag	2*20 m 6*6 m	50 m, 100 m and 200 m freestyle	6
Dragunas et al. (49)	Young swimmers (19.36)	5	9 per week	YES	Water resisted training	Drag suit–trained	3*50 m 4*25 m	50 m freestyle	6
Ravé et al. (50)	Young male swimmers (16.22 ± 2.63)	6	5 per week	YES	Water resistance exercises	Power rack	50–70% 1RM	50 m crawl 50 m competition-style time trials	5
Gourgoulis et al. (51)	Young female swimmers (13.08 ± 0.9)	11	6 per week	YES	Water resistance exercises	Water parachute	6*15 m 4*25 m	50 m crawl 100 m crawl 200 m crawl	5
Kojima et al. (52)	Young Swimmers (13.6 ± 1.1)	10	2 per week	YES	Water resistance exercises	10 m sprints with progressively increasing resistance	70–80% 1RM	50 m freestyle	5
Papoti et al. (53)	Young swimmers (16.0 ± 2.1)	11	6 per week	YES	Water resistance exercises	A 6 m elastic cord was connected to a load cell	70–90% 1RM	Free-swimming 200 m, 100 m, and 400 m	5
Keiner et al. (54)	Young swimmers (17.5 ± 2)	2	2 per week	YES	Land strength training	Back squat dead lift started both arms sit-up	1RM	15–100 meters in freestyle, breaststroke and backstroke	5
Salman et al. (55)	Young swimmers	8	2 per week	YES	Water resistance exercises	Parachute gloves	Did not show	100 m freestyle	4

*1RM, 1 Repetition maximum*.

## Results

### Participant Characteristics

Table 2 summarizes the characteristics of the participants from the 16 papers who meet the criteria for inclusion in this review. In total 338 participants, of which 224 were males and 91 were females, were collected where participants had an age range of between 11 and 20 years old (15, 33, 42–55). The pre-test difference between the control and experimental groups was minimal, in which 15 studies involved swimmers who were trained for national or international competitions (15, 33, 42–54). One study, however, did not mention the swimming capabilities of the athletes (55).

### Study Design and PEDro Score

The research design and PEDro scores were also summarized in Table 2. In total 10 studies reported an intervention duration of 8 to 12 weeks (15, 33, 43, 45, 48, 51–55) with the longest duration reported at 24 weeks (44) There were 14 studies that used randomization (15, 33, 42, 43, 45) in their design while the remaining 2 studies did not randomly assign the participants into intervention conditions (44, 47). The PEDro scores for all studies were 4 (55), 5 (50–54) and 6 (15, 33, 42–49). All studies were deducted points for items related to blindness in participants, therapists, and evaluators. The differences in scores were mainly based on whether the study involved random assignment.

### Resistance Training and Strength Training

Through these 16 papers, it was found that some articles adopted the term “resistance training” while others used the term “strength training.” Resistance training emphasized the way that muscle tissues resisted force, generally including combined equipment, free-weight training and self-weight training. The equipment necessary were: isokinetic dynamometer, Swiss ball, solid ball, bungee rope, drag parachute and drag hand webs (15, 33, 42, 43, 48–53, 55). The other articles used “strength training” that highlighted the purpose of training, which is to increase muscle strength. The equipment required include resistance parachute, elastic rope, resistance suit, isokinetic dynamometer, barbell and dumbbells (44, 45, 47, 54). While there were no noticeable differences in concept and training equipment for these terms, their primary focuses were different.

### Training Programmes

Table 2 summarizes training types, content, arrangements and test items related to strength training intervention and training used by swimmers during the study period. Resistance training was mainly divided into water resistance training (42, 43, 48–53, 55) and land resistance training (15, 33, 44–47, 54). Water training mainly included resistance parachutes, gloves, tie ropes and resistance clothes, whereas land training comprised of rubber bands, Swiss balls, solid balls, sit-ups, dumbbells, push-ups, and large resistance equipment. However, the resistance training was mainly at low intensities, and the outcome measures were primarily either through freestyle or short distances. All research used only one type of resistance training, which were found to be superior to traditional strength training. Interestingly, none of the studies examined the effect of water resistance training in combination with land resistance training on swimming performance (15, 33, 42–55).

The intensity of training was also summarized in Table 2. Six articles showed the strength of movement in percentage, with the passion ranging from 30–40% 1RM (47) to 80–90% 1RM (15), while the intensity of other training programmes ranged from 50–80% 1RM (50, 52–54). The training intensity did not reach 100% 1RM. Resistance training in water had a specific resistance, covered a short swimming distance of 20–30m (48, 49, 51) and lasted less than 30 seconds (42, 43). Therefore, these resistance training were low-intensity and high-speed. Each resistance training conducted on land was performed more than 30 times (33, 45, 46) and only low-intensity resistance was possible with more repetitions and faster natural speed.

### Strength Outcomes

In the 16 studies in Table 3, the improvement in swimming performance were significant in both water and land resistance training (15, 33, 42, 45, 46, 48–54). One study showed that muscle strength and swimming performance were highly correlated (44). Another study demonstrated that two types of water resistance training were more effective in improving swimming performance than one type of training (55). In addition, research showed that water resistance training improved swimmers' performance more than traditional land strength training, but land resistance training was more effective than conventional land strength training. Furthermore, strength training on land also improved the performance of swimmers. With the swimming performance of adolescents improving especially at 50 m and 100 m distances, programme should use the low-intensity, high-speed force approach to engender results among youths (15, 42, 45–48, 51, 52, 54, 55).

**Table 3:** Group, main outcomes and participant characteristics.

**Study**	**Group**	**Main outcomes**	**Participant characteristics**			
			**(I/C)**	**Sex**	**Age (years)**	**Training background**
Huang (42)	1. Water resistance traction. 2. Conventional training regimen.	Underwater resistance training can effectively improve swimming performance	16	8 =F 8 = M	13	National level 2
Zhao (33)	1. On shore resistance training. 2. Traditional strength exercise.	The training effect of the land resistance training is higher than conventional strength training.	20	M	11–12	National level 2
Batalha et al. (43)	1. The water group. (WG). 2. The land group. (LG)	Dry-land training is more effective than water training.	25	M	12–15	Three years of experience
Dalamitros et al. (44)	1. Dr—land strength training. 2. Swimming training.	Bilateral muscle strength deficit and knee F/E peak torque ratio only reported small changes	11	M	14.82 ± 0.45	National level
Amaro et al. (45)	1. Swim training alone. 2. Dry-land programme based on sets. 3. Dry-land focused on explosiveness.	Dry-land S&C training may lead to an improvement in dry-land strength.	21	M	12.7 ± 0.7	Competitive swimmers
Naczk et al. (46)	1. Dry-land strength training. 2. Swimming training.	There is a marked improvement in swimmers' performance in dry-land.	14	12 = M 14 = F	15.8 ± 0.4	National level
Marques et al. (47)	1. Male athletes. 2. Female athletes.	Improved swimming performance, with no significant difference between the two sexes.	10	5 = M 5 = F	16.6 ± 0.7	International level
Girold et al. (15)	1. Dry-land strength. 2. Water resistance. 3.Traditional swimming training.	Dry-land strength or water resistance is more effective than using traditional swimming training methods alone.	21	10 = M 11 = F	16.5	National level
Toussaint et al. (48)	1. A training group 2. A control group	The POP is a specific training device especially suitable for increasing maximal power output during swimming.	22	16 = M 6 = F	18.50 ± 3.30	National level
Dragunas et al. (49)	1. Control group 2. Drag suit–trained group	The stroke speed of the resistance suit training group was significantly improved.	18	10 = M 8 = F	19.36	National level
Ravé et al. (50)	1. A standard training group (GS) 2. A pattern group (GP)	In the GP events, the 50m freestyle improved.	16	M	16.22 ± 2. 63	National level
Gourgoulis et al. (51)	1. Water parachute resistance training 2. Resistance training was not increased	Improvements were only significant in the experimental group.	12	F	13.08 ± 0.9	National level
Kojima et al. (52)	1. Resisted sprint swim training 2. Non-resisted sprint swim training	A boycott of sprint training is no more effective than a boycott of sprint training.	24	12 = M 12 = F	13.6 ± 1.1	Well-trained
Papoti et al. (53)	1. Tethered Resistance Training 2. Traditional freestyle training	The tethered resistance training method helps to improve the performance of swimmers.	34	22 = M 12 = F	16.0 ± 2. 1	Least 2 years
Keiner et al. (54)	5 groups (each with 4 or 5 subjects)	The maximal strength parameters of the upper and lower extremities and maximal trunk strength are good predictors of performance in sprint swimming in trained adolescent swimmers in different disciplines.	21	12 = M 9 = F	17.5 ± 2	National level
Salman et al. (55)	1. Trained using the umbrella resistance. 2. Trained using paw resistance. 3.Two types of resistance.	Two kinds of resistance combined training, improve swimming performance is more effective.	9	NO	NO	NO

## Discussion

This systematic review aimed to examine the influence of resistance training on the performance of adolescent swimmers and summarize resistance training methods for these swimmers. According to the data collected, resistance training can directly impact performance with proper training being beneficial to adolescent growth and development. Appropriate resistance training can help increase muscle strength, promote body growth and prevent injuries (57). To achieve the best training effect in adolescent swimmers, a low-intensity, high-speed force resistance training programme is recommended for optimal training results. However, there were inconsistencies in the literature, which can be attributed to differences in methodology and participant demographics; hence, practitioners should apply the recommendation in this study to their athletes with caution. The most significant concern with resistance training in children and adolescents was the risk of injury due to the excessive use of soft tissues, especially for the lower back, back and shoulders (58). If a resistance exercise programme was designed to exceed a child's capacity, the pleasure in exercising may be diminished through the increase of acute or overtraining injuries (59). Moreover, although the PEDro scores were moderate (i.e. 4, 5 or 6), the quality of the work reviewed in this article were generally acceptable considering the inevitable restrictions imposed by training intervention studies related to blindness.

### Dry-Land Resistance Training Modalities

Some studies confirmed that dry-land strength training can improve swimming performance (15, 33, 44–47, 54). Therefore, widespread dry-land strength training procedures were reported by swimming athletes to improve performance in competitions. In addition, studies showed the auxiliary effect of kicking in improving swimming speed (60–62). In general, dry-land resistance training includes two types of strength training which were basic and special. Basic strength training comprised of (1) bench press barbell, which was to develop the maximum muscle strength of the pectoralis major, triceps and forearm muscles, (2) dumbbell exercise to develop the strength of pectoralis major and latissimus dorsi, and (3) Swiss ball and medicine ball to build local muscles. The Swiss ball can improve the control ability of the whole body muscles, help to develop stability and maintain a good body posture during swimming (33, 44, 45). The techniques for special strength training included isokinetic tension, pulley tension and isokinetic dynamometer. The isokinetic pulling force is divided into three positions: prone, supine and standing. This exercise mirrors one's posture in water, introduces force on one or both arms and imitates the movement and resistance in water to a great extent (15, 33, 44–46, 54).

### Underwater Resistance Training Modalities

Some studies confirmed that underwater resistance training can improve swimming performance (42, 43, 48–53, 55). “Underwater” Resistant Sprint Swimming Training (RST) was developed to increase the possibility of effective transfer of mature swimmers (14, 28, 63). Generally, water resistance training included the usage of a variety of equipment to increase the swimming resistance of athletes, namely (1) power frame, (2) rubber tension, (3) Increase water resistance, elastic rope, and (4) parachutes, gloves and resistance clothes (49, 51, 55). Power frame is a traditional method used to improve swimming intensity (52). On the other hand, rubber tension involves tying the two ends to the waists of each athlete during two-person resistance training Another variation of rubber tension involves one end of the rubber tension being tied to the waist of two athletes, while the other end being tied to the athlete on the pool wall (42, 43). Furthermore, with an elastic rope, one end is connected to a load cell while the other end is connected to the swimmer's waist (48, 50, 55).

### Swimming Performance

Table 2 summarized the main outcomes of the 16 studies selected. Multiple studies found that adolescent swimmers who underwent dry-land resistance training (15, 33, 43, 45, 46, 52) and water-resistance training (15, 42, 48, 49, 51, 53, 54) reported significant improvements in their swimming performance as compared to traditional swimming training. The swimming performances studied in most studies were mainly freestyle, specifically 50 m freestyle (15, 42, 45–52, 54), 100 m freestyle (33, 48, 51, 53–55), 200 m freestyle (48, 51, 53), and 400 m freestyle (42, 53). Other strokes that were measured as swimming performance were 100 m breaststroke (33), 100 m backstroke (33), and 100 m butterfly (33, 46). One study, however, found that resistance training showed little change in bilateral muscle strength and F/E peak torque ratio of the knee (44). Moreover, competitive swimming races consist of four different segments: start, clean, turn and finish (64, 65). As the race distance becomes longer (e.g. from 50 m to 1,500 m), different phases during the swim had different contributions to the final race time. For example, in short 100 m events, the start and turn accounted for nearly one-third of the final race time (66). Therefore, these phases are crucial for the impact of swimming performance. More concerning is the limited studies that have investigated the effect of resistance training specifically on the start and turn skills of adolescent swimmers especially for a 50 m event.

## Limitations

Overall, this review provides substantial evidence of considerable quality and the beneficial effects of different resistance training programs on swimming performance. However, this review has several limitations. However, there are several limitations to this review. First, most of the studies involved unequal numbers of male or female, either only male or only female. If present, it could be important because there are differences in assessing swimming performance based on sex, which could affect the final study results. Second, none of the studies in this review stated sample size calculation methods. Determining the sample size is influenced by several factors, including the purpose of the study, the size of the population, the risk of selecting the sample, and the allowable sampling error. Therefore, inappropriate, insufficient, or excessive sample size can affect quality and accuracy. Errors in the calculation of sample size in included studies may affect study results. Third, most studies did not record or control for the exercises that participants performed outside of the research setting. Therefore, it is difficult to predict the effect of resistance on swimming performance of athletes.

## Conclusion and Future Research

Maglischo's theory of resistance training in swimming posited that there are two types of resistance training that can improve swimming performance, which are training conducted in water and on land (1). Existing literature also showed that underwater resistance training (42, 43, 48–53, 55) and terrestrial resistance training (15, 33, 44–47, 54) can improve the swimming performance of adolescents. At present, no study on the effects of combined land and water resistance training on swimming performance has been reported. Furthermore, while the improvements in the overall performance of teenage swimmers undergoing resistance training were noticeable, no research detailing the impact of specific techniques on performance were conducted.

This review is the most comprehensive review to date relating to the effects of resistance training on the performance of young swimmers. Numerous studies found that resistance training can improve swimming performance in response to all resistance training programmes. Based on this review, the strength training programme recommended for young swimmers is a low-intensity, high-speed programme. Furthermore, resistance training interventions were mainly short distances of between 50–100 meters. Future research may consider investigating the effects of resistance training on adolescents' start and turn skills. In addition, the impact of combining land and water resistance training on teenage swimmers warrants further study.

## Data Availability Statement

The datasets presented in this study can be found in online repositories. The names of the repository/repositories and accession number(s) can be found in the article/supplementary material.

## Author Contributions

KS and WG contributed to the design, acquisition, analysis, interpretation of the data for the systematic review, drafted the work, revised it critically for important intellectual content, approved the version to be published, and agreed to be account-able for all aspects of the work ensuring that the questions related to the accuracy or integrity of any part of the work are appropriately investigated and resolved. All authors contributed to the article and approved the submitted version.

## Conflict of Interest

The authors declare that the research was conducted in the absence of any commercial or financial relationships that could be construed as a potential conflict of interest.

## Publisher's Note

All claims expressed in this article are solely those of the authors and do not necessarily represent those of their affiliated organizations, or those of the publisher, the editors and the reviewers. Any product that may be evaluated in this article, or claim that may be made by its manufacturer, is not guaranteed or endorsed by the publisher.
